# Supplementary material for: Allelopathic Effects of Artemisia thuscula and Plocama pendula on the Invasive Plant Cenchrus setaceus and Crops
Source: Plants (Basel). 2025 Oct 14;14(20):3159. doi: 10.3390/plants14203159 (PMC12567509; doi:10.3390/plants14203159)
Supplement: Supplementary file 1 [file plants-14-03159-s001.zip › plants-3836011-supplementary.pdf]

Supplementary Information

Allelopathic Effects of *Artemisia thuscula* and *Plocama pendula* on the Invasive Plant *Cenchrus setaceus* and Crops

Ana Fuvel [1,2], Andreea Cosoveanu [3,4,*], Jorge Sopena Lasala [3], José Ramón Arévalo [3] and Raimundo Cabrera [3]

[1] Agroecology and Environment Research Unit, Institut Supérieur d'Agriculture Rhône-Alpes (ISARA), 23 Rue Jean Baldassini, 69364 Lyon, France; anamfuvel@gmail.com
[2] Department of Plant Sciences, Faculty of Biosciences, Norwegian University of Life Sciences, NO-1432 Ås, Norway
[3] Department of Botany, Ecology and Plant Physiology, Faculty of Sciences, Section Biology, Universidad de La Laguna, 38206 La Laguna, Tenerife, Spain; jsopenal@ull.edu.es (J.S.L.); jarevalo@ull.edu.es (J.R.A.); rcabrera@ull.edu.es (R.C.)
[4] Gabinete de Estudios Ambientales (GEA), S.L.U, 38659 Tacoronte, Tenerife, Spain

* Correspondence: acosovea@ull.edu.es

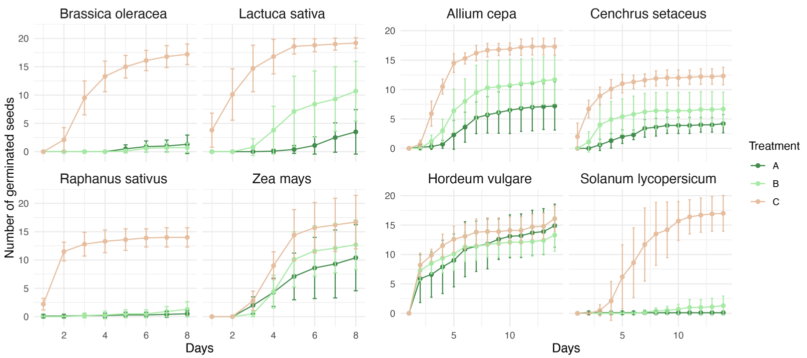

Figure S1. Germination dynamics for Group 1 (left) and Group 2 (right) species, showing means and standard deviations values in presence of treatment with *A. thuscula* (A), *P. pendula* (B) and control (C) under growth chamber conditions.

Table S1 Germination indices mean ± sd values per treatment (A = *A. thuscula,* B = *P. pendula* and C= control) for all species under growth chamber conditions

Species	Treatment	FPG	MGT	GI	FDG	LDG
	A	36.0 ± 20.39	6.84 ± 1.30	58.7 ± 33.52	4.2 ±1.62	10.00 ± 3.02
A. cepa	B	53.5 ± 25.61	6.18 ± 0.85	103.9 ± 43.68	3.2 ± 0.42	11.30 ± 2.67
	C	86.5 ± 7.09	4.42 ± 0.49	182.6 ± 11.55	2.40 ± 0.52	8.80 ± 2.35
	A	6.50 ± 4.71	5.89 ± 0.69	4.0 ± 4.71	5.33 ± 0.52	6.33 ± 1.03
B. oleracea	B	3.50 ± 2.51	6.00 ± 0.35	2.1 ± 2.52	5.80 ± 0.45	6.20 ± 0.45
	C	86.0 ± 12.66	3.77 ± 0.43	90.0 ± 12.66	2.40 ± 0.52	6.80 ± 1.03
	A	21.0 ± 13.12	6.08 ± 1.59	37.1 ± 13.11	4.00 ± 1.05	8.80 ± 3.43
C. setaceus	B	30.5 ± 38.09	4.50 ± 1.29	72.9 ± 38.09	2.70 ± 0.67	8.20 ± 3.39
	C	61.0 ± 15.07	3.08 ± 0.61	146.3 ± 15.07	1.50 ± 0.58	8.30 ± 3.27
	A	74.5 ± 47.43	5.43 ±1.54	144.9 ± 47. 43	2.00 ± 0.00	12.10 ± 2.64
H. vulgare	B	66.5 ± 28.78	3.93 ± 1.20	143.7 ±28. 78	2.00 ± 0.00	9.80 ± 3.74
	C	80.5 ± 23.03	4.37 ± 0.80	170.7 ± 23.03	2.00 ± 0.00	12.30 ±2.69
	A	17.5 ± 19.61	6.83 ± 0.63	7.7 ± 8.49	5.71 ± 1.38	7.43 ± 0.79
L. sativa	B	53.5 ± 26.36	5.76 ± 1.20	40.1 ± 27.32	4.40 ±1.58	7.80 ± 0.42
	C	96.0 ± 4.59	2.73 ± 0.73	121.0 ± 16.52	1.10 ± 0.32	5.80 ± 1.62
	A	2.50 ± 2.64	4.80 ± 2.86	2.2 ± 2.90	4.80 ± 2.86	4.80 ± 2.86
R. sativus	B	6.50 ± 6.69	5.72 ± 1.72	3.7 ± 3.96	5.00 ± 1.79	6.50 ±2.07
	C	70.0 ± 8.50	2.18 ± 0.14	95.5 ± 12.32	1.00 ± 0.00	4.50 ± 1.08
	A	0.50 ±1.58	2.00 ± NA	1.3 ±4.11	2.00 ± NA	2.00 ± NA
S. lycopersicum	B	6.50 ± 8.18	9.72 ± 2.43	6.2 ± 7.71	9.00 ± 2.68	10.50 ± 2.26
	C	84.0 ±14.87	6.88 ± 1.57	139.5 ± 42.81	4.30 ± 1.06	9.90 ± 2.38
	A	49.0 ± 30.89	4.50 ± 0.66	41.8 ± 23.30	3.00 ± 0.00	6.90 ± 1.37
Z. mays	B	63.5 ± 21.99	5.02 ± 0.48	51,4 ± 20.03	3.50 ± 0.53	6.90 ± 1.10
	C	83.5 ± 23.58	4.46 ± 0.45	74.8 ± 21.05	3.10 ± 0.32	6.70 ± 1.34

Germination indices were calculated at the end of the germination trials on growth chamber (i.e after 8 days for *Z. mays, L. sativa, B. oleracea* and *R. sativus*; and after 14 for *A. cepa, S. lycopersicum, H. vulgare* and *C. setaceus)*

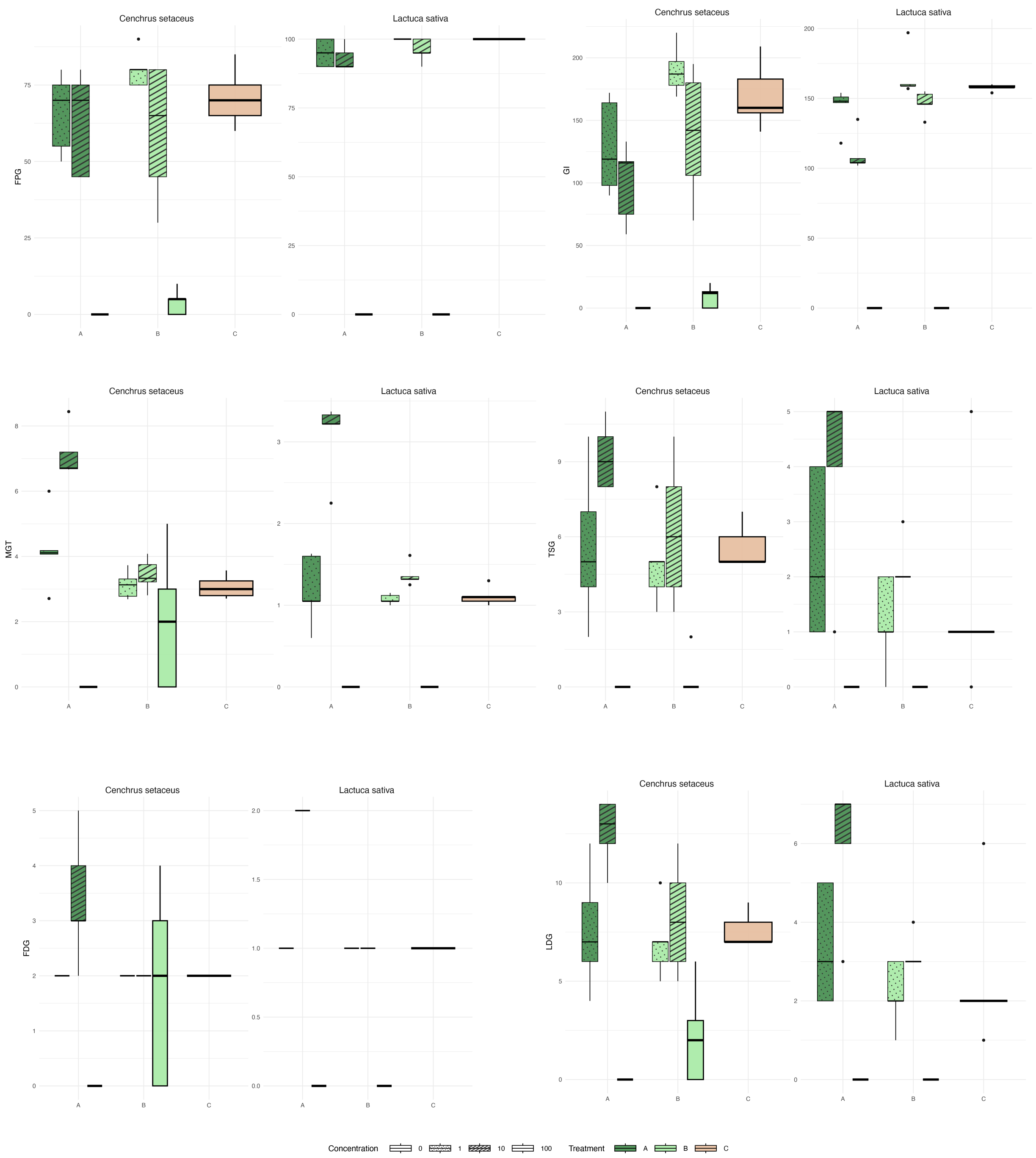

Figure S2 Germination response of species to treatments (*A. thuscula* – A, *P. pendula* – B, control – C) at three concentrations (100%, 10% and 1%) under growth chamber conditions. Final Percentage of Germination (FPG), Germination Index (GI), Mean Germination Time (MGT), First Day of Germination (FDG) and Last Day of Germination (LDG) shown as boxplots (left to right, top to bottom).

Table S2 Germination indices mean ± sd values per treatment (A = *A. thuscula,* B = *P. pendula* and C= control) for all species in the greenhouse assays

Species	Treatment	FPG	MGT	GI	FDG	LDG
A. cepa	A	37.88 ± 31.96	7.46 ± 1.99	37.42 ± 34.86	7.19 ± 2.48	8.54 ± 2.43
	B	28.79 ± 33.02	8.92 ± 2.58	22.20 ± 31.70	7.72 ± 2.54	9.72 ± 2.79
	C	41.03 ± 32.69	8.53 ± 2.50	34.42 ± 36.61	7.75 ± 2.96	9.75 ± 2.31
B. oleracea	A	45.45 ± 29.02	6.19 ± 1.54	9.86 ± 10.86	5.82 ± 1.61	6.55 ± 1.65
	B	23.74 ± 23.94	5.75 ± 1.64	6.36 ± 9.93	5.67 ± 1.74	5.87 ± 1.59
	C	59.09 ± 30.83	5.24 ± 1.16	17.86 ± 12.93	4.46 ± 1.39	6.03 ± 1.55
C. setaceus	A	21.21 ± 30.18	7.12 ± 1.50	22.97 ± 35.36	6.63 ± 1.52	7.85 ± 2.18
	B	22.22 ± 25.04	7.03 ± 2.61	25.94 ± 33.23	6.97 ± 2.68	7.17 ± 2.61
	C	53.03 ± 28.03	5.59 ± 1.37	79.55 ± 48.40	4.95 ± 1.25	6.29 ± 2.05
H. vulgare	A	11.11 ± 17.86	6.20 ± 2.95	15.14 ± 26.06	5.95 ± 3.00	6.45 ± 3.30
	B	11.61 ± 18.94	6.18 ± 1.55	15.48 ± 26.20	5.90 ± 1.62	6.55 ± 1.88
	C	11.11 ± 17.86	5.65 ± 2.35	17.15 ± 29.61	5.40 ± 2.23	5.90 ± 2.92
L. sativa	A	63.13 ± 24.90	5.78 ± 1.11	16.73 ± 13.50	5.31 ± 1.24	6.31 ± 1.27
	B	51.51 ± 32.12	4.95 ± 1.50	18.27 ± 15.27	4.31 ± 1.61	5.59 ± 1.80
	C	83.84 ± 20.46	3.44 ± 0.78	47.92 ± 16.74	2.77 ± 0.63	4.45 ± 3.10
R. sativus	A	68.18 ± 33.81	5.29 ± 1.28	25.67 ± 30.12	4.53 ± 1.69	6.05 ± 1.25
	B	29.29 ± 27.12	5.83 ± 1.66	7.42 ± 9.93	5.45 ± 1.90	6.17 ± 1.70
	C	77.77 ± 24.34	4.23 ± 1.12	34.36 ± 15.28	3.41 ± 1.15	5.24 ± 1.57
S. lycopersicum	A	47.98 ± 33.14	11.43 ± 1.97	16.62 ± 16.81	10.39 ± 2.48	11.98 ± 2.51
	B	65.66 ± 35.07	10.75 ± 1.93	26.14 ± 19.50	9.61 ± 2.17	11.44 ± 1.80
	C	92.93 ± 14.92	7.80 ± 1.31	87.55 ± 31.60	6.77 ± 1.45	8.76 ± 1.87
Z. mays	A	47.47 ± 34.62	6.10 ± 1.11	12.67 ± 18.97	5.65 ± 1.31	6.65 ± 1.25
	B	57.07 ± 30.81	6.08 ± 1.18	11.65 ± 10.13	5.44 ± 1.48	6.68 ± 1.27
	C	78.28 ± 28.94	5.38 ± 1.32	24.08 ± 17.51	4.67 ± 1.65	6.08 ± 1.30

Germination indices were calculated at the end of the germination trials on greenhouse (i.e after 8 days for *Z. mays, L. sativa, B. oleracea* and *R. sativus*; and after 14 for *A. cepa, S. lycopersicum, H. vulgare* and *C. setaceus)*

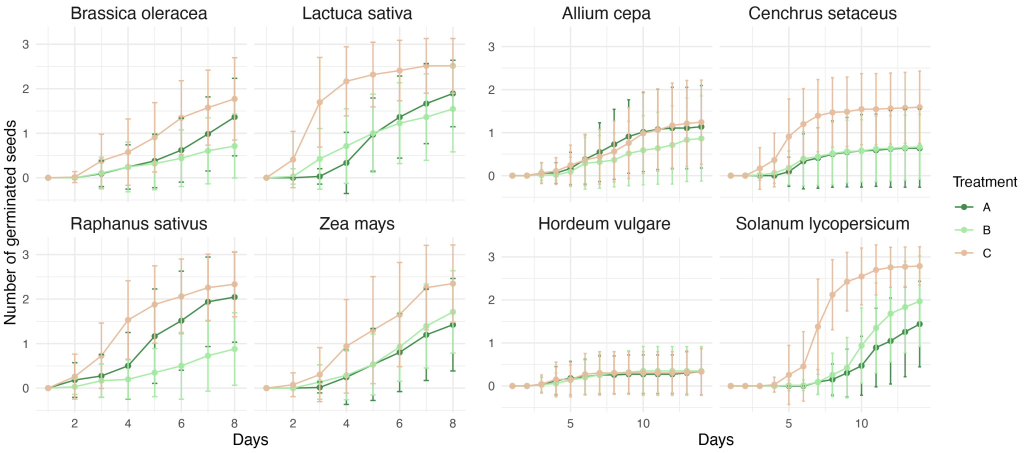

Figure S3 Germination dynamics for Group 1 (left) and Group 2 (right) species, showing means and standard deviations values in presence of treatment with *A. thuscula* (A), *P. pendula* (B) and control (C) under greenhouse conditions.

Table S3 FPG mean ± sd values per species and treatment (A = *A. thuscula*, B = *P. pendula* and control with halved value), under growth chamber and greenhouse assays (up-scaled trial)

Species	Growth Chamber			Greenhouse		
	Treatment A	Control / 2	Treatment B	Treatment A	Control / 2	Treatment B
A. cepa	36.0 ± 20.39a	43.3 ± 3.55a	53.5 ± 26.61a	37.9 ± 31.96a	20.5 ± 16.3b	28.8 ± 33.0ab
B. oleracea	6.5 ± 4.71a	43.0 ± 6.33b	3.5 ± 2.51a	45.5 ± 29.02a	29.5 ± 15.41b	23.7 ± 23.9c
H. vulgare	74.5 ± 47.43a	40.3 ± 11.52b	66.5 ± 28.78a	11.1 ± 17.86a	5.56 ± 8.93a	11.6 ± 18.9a
L. sativa	17.5 ± 19.61a	48.0 ± 2.30b	53.5 ± 26.36ab	63.1 ± 24.90a	41.9 ± 10.23b	51.5 ± 32.1ab
R. sativus	2.5 ± 2.64a	35.0 ± 4.25b	6.5 ± 6.69a	68.2 ± 33.8a	38.9 ± 12.17b	29.3 ± 27.1c
S. lycopersicum	0.5 ± 1.58a	42.0 ± 7.44b	6.5 ± 8.18a	48.0 ± 33.1a	46.5 ± 7.46a	65.7 ± 35.1b
Z. mays	49.0 ± 30.89a	41.8 ± 11.79a	63.5 ± 21.99ab	47.5 ± 34.6a	39.1 ± 14.47a	57.1 ± 30.8ab

Different letters represent significant differences between lixiviates and control groups ($p < 0.05$)

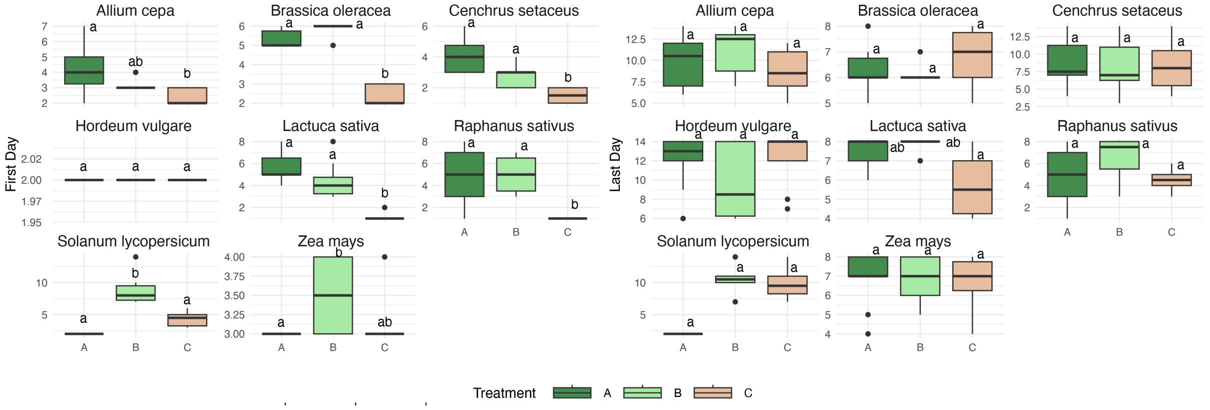

Figure S4 Germination response of species to treatments (*A. thuscula* – A, *P. pendula* – B, control – C) under growth chamber conditions. First Day of Germination (FDG) and Last Day of Germination (LDG) shown as boxplots (left to right). Different letters indicate treatment differences ($p < 0.05$).

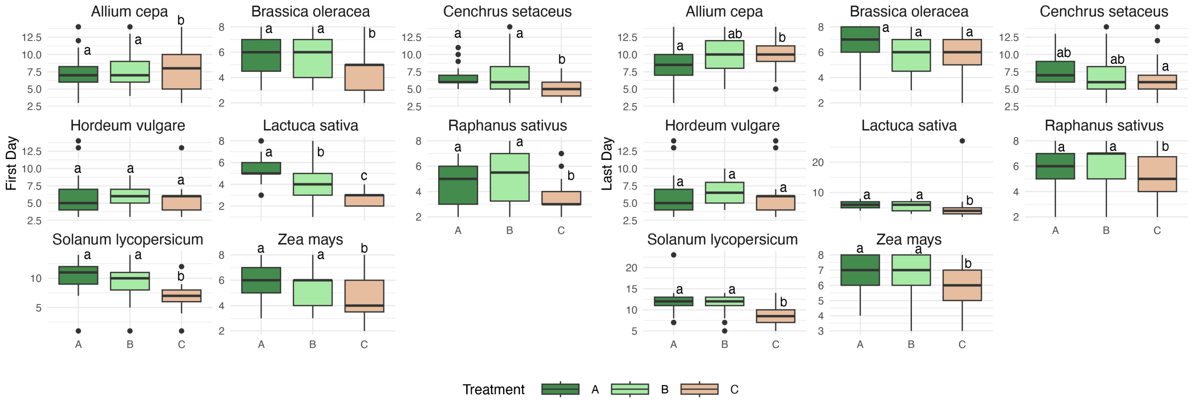

Figure S5 Germination response of species to treatments (*A. thuscula* – A, *P. pendula* – B, control – C) under greenhouse conditions. First Day of Germination (FDG) and Last Day of Germination shown as boxplots (left to right). Different letters indicate treatment differences ($p < 0.05$).

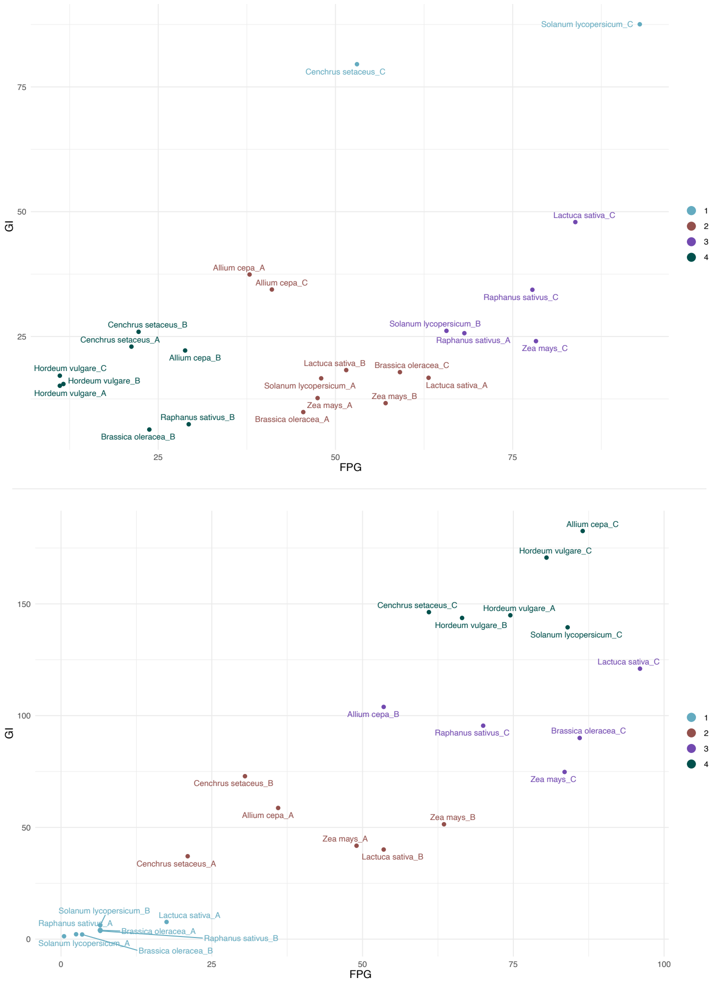

Figure S6 Species clustering by germination sensitivity to *A. thuscula* (A), *P. pendula* (B) and control (C) treatments, under growth chamber conditions (up) and greenhouse conditions (bottom). Clusters reflect differences in Final Percentage of Germination (FPG) and Germination Index (GI). *Note: y-axis scales differ between panels to preserve plot resolution and facilitate visual interpretation of clustering patterns.*
